# Extracorporeal membrane oxygenation in aluminum phosphide poisoning in Nepal: a case report

**DOI:** 10.1186/s13256-018-1864-z

**Published:** 2018-10-28

**Authors:** Achyut Sharma, Apurb Sharma, Anil Acharya, Diptesh Aryal, Bijoy G. Rajbanshi, Prajjwal Raj Bhattarai, Ashim Regmi, Anup Ghimire

**Affiliations:** 1 Department of Anaesthesia, Pain Management, and Critical Care, Nepal Mediciti Hospital, Nakhkhu Patan, Karyabinayak 44600 Nepal; 2 Department of Cardiothoracic and Vascular Surgery, Nepal Mediciti Hospital, Nakhkhu Patan, Karyabinayak 44600 Nepal

**Keywords:** ECMO, Extracorporeal membrane oxygenator, Aluminum phosphide poisoning, Poisoning in Nepal, Veno-arterial ECMO

## Abstract

**Background:**

Aluminum phosphide is a very common suicide agent in developing countries like Nepal. Due to the unavailability of a specific antidote, mortality is very high because the phosphine molecule that is formed leads to inhibition of the cytochrome oxidase enzyme system in mitochondria. Extracorporeal membrane oxygenation provides life-saving support to the cardiovascular and respiratory systems until the deadly poison is eliminated from the body.

**Case presentation:**

We encountered one case of 67-year-old Asian woman, a known case of major depressive disorder, who presented to our center with suicidal ingestion of aluminum phosphide with cardiovascular and respiratory dysfunction. On presentation in our emergency room, she had an ejection fraction of 20% and had to be immediately intubated for respiratory failure. Based on the evidence of almost 100% mortality with aluminum phosphide poisoning, extracorporeal membrane oxygenation was initiated in our intensive care unit. Her general condition and hemodynamics gradually improved over the course of 2 days and she was weaned from extracorporeal membrane oxygenation and ventilator by post-extracorporeal membrane oxygenation days 3 and 4, respectively. After psychiatric evaluation and establishment of normal vital parameters, she was moved out of intensive care unit on post-extracorporeal membrane oxygenation day 6 and discharged to home on post-extracorporeal membrane oxygenation day 10.

**Conclusions:**

Although this seems to be a small step in terms of global perspective, it is a giant stride for a developing country. The management of reversible but severe cardiac and respiratory failure certainly opens up newer scopes where we can ensure a quality health care service being made accessible even to the most underprivileged people.

## Background

Aluminum phosphide (AlP) ingestion or inhalation by accident or with suicidal intent is one of the commonest modes of poisoning in Nepal [1, 2]. This lethal form of poisoning has no specific antidote and has a mortality rate as high as 45 to 100% [3, 4]. Although a majority of patients may remain asymptomatic or have mild symptoms of nausea and vomiting on initial presentation, there is rapid deterioration in clinical condition mainly due to cardiac and respiratory failure. The formation of phosphine (PH_3_) gas when AlP interacts with moisture in the environment or acid in the stomach causes a cyanide-like toxicity in which mitochondria are unable to utilize oxygen due to the inhibition of the cytochrome oxidase enzyme system. The resultant anaerobic metabolism and severe lactic acidosis causes multiorgan dysfunction and death ensues rapidly. A variety of supportive measures in the form of rapid and prompt lavage with potassium permanganate, use of activated charcoal to delay absorption, magnesium sulfate, vegetable oils, coconut oil [5, 6], and so on have been tried with mixed results. A therapy that provides astounding support to the organ system until the dreaded poison is eliminated via the kidneys is extracorporeal membrane oxygenation (ECMO); ECMO has shown consistently positive results in the management of AlP poisoning in several countries. Although ECMO has been used in Nepal previously [7], this case report describes probably the first use of ECMO in an A1P case in Nepal. This case opens up a whole new horizon in the management of one of the commonest causes of fatal rodenticide poisoning in south asia.

## Case presentation

A 67-year-old Asian woman with a known long history of major depressive disorder was brought to our emergency room (ER) with complaints of intentional ingestion of two tablets of AlP 5 hours prior to presentation. Except for the depressive illness for which she was taking a tablet form of escitalopram 20 mg twice daily, with which she was compliant, there was no other significant medical, surgical, or family history. She was seen by her son who found her in her room complaining of nausea and vomiting when she expressed to him about her ingesting the tablets. She had multiple episodes of vomiting containing greenish-colored particulate material which was not blood mixed. She also had three episodes of loose stools and generalized body weakness with altered sensorium. Before she was brought to our ER, she was taken to another center where she was primarily managed with gastric lavage and initial resuscitation. When she was evaluated in our ER, she was drowsy, her Glasgow Coma Scale (GCS) was 8/15, and her pupils were bilaterally 4 mm and sluggish reactive to light. Her pulse was 58/minute, she had blood pressure (BP) of 80/60 mmHg, her respiratory rate (RR) was 35/minute, and arterial oxygen saturation was 93% with supplemental oxygen via face mask. On auscultation, there were crepitations bilaterally along with decreased breath sounds. An initial arterial blood gas (ABG) done in ER revealed pH of 7.094, partial pressure of oxygen in blood (PaO_2_) 130 mmHg, partial pressure of carbon dioxide in blood (PaCO_2_) 23.5 mmHg, bicarbonate (HCO_3_) 8.9 mmHg, and serum lactate of 15.

Besides these clinical findings and laboratory parameters other tests were within normal range. A chest X-ray was done immediately, which showed infiltrations with bilateral opacities. A 12-lead electrocardiogram (ECG) showed sinus rhythm, non-specific ST-T changes in all leads. Cardiologists were immediately called for screening echocardiographic evaluation which showed significantly reduced systolic function of left ventricle with an ejection fraction (LVEF) of 20%. While evaluation was ongoing, a wide bore canula was opened, and dopamine was started to support the blood pressure. However, patient’s condition continued to deteriorate and an immediate plan for veno-arterial ECMO was made. After obtaining consent for ECMO from patient’s son, the intensive care unit (ICU) team was alerted and cardiac surgeons were informed accordingly. The patient was successfully connected to ECMO (Sorin SCPC Centrifugal Pump System) via right femoral vein and femoral artery. An extra 6F sheath was inserted distally into femoral artery for perfusion of distal leg.

In the immediate postoperative period the patient was kept on mechanical ventilator volume assist-control mode on low tidal volume lung protective ventilation at 40% fraction of inspired oxygen concentration (FiO_2_) with positive end-expiratory pressure (PEEP) of 7 cmH_2_O. We were able to deliver an almost constant flow of 3 L/minute/m^2^ on ECMO and provided oxygenation at FiO_2_ of 70%. She was also kept on epinephrine, norepinephrine support, magnesium sulfate, vitamin C, thiamine, hydrocortisone, heparin infusion, and sodium bicarbonate therapy. An ABG done immediately after the procedure showed improvement in acid-base status as well as decrease in lactate level to 0.31. Blood gases were repeated 4 hourly. Boluses of heparin 1000–3000 U were given along with adjustment in background infusion rate to keep the activated clotting time (ACT) between 180 and 220 seconds.

**Fig. 1:**
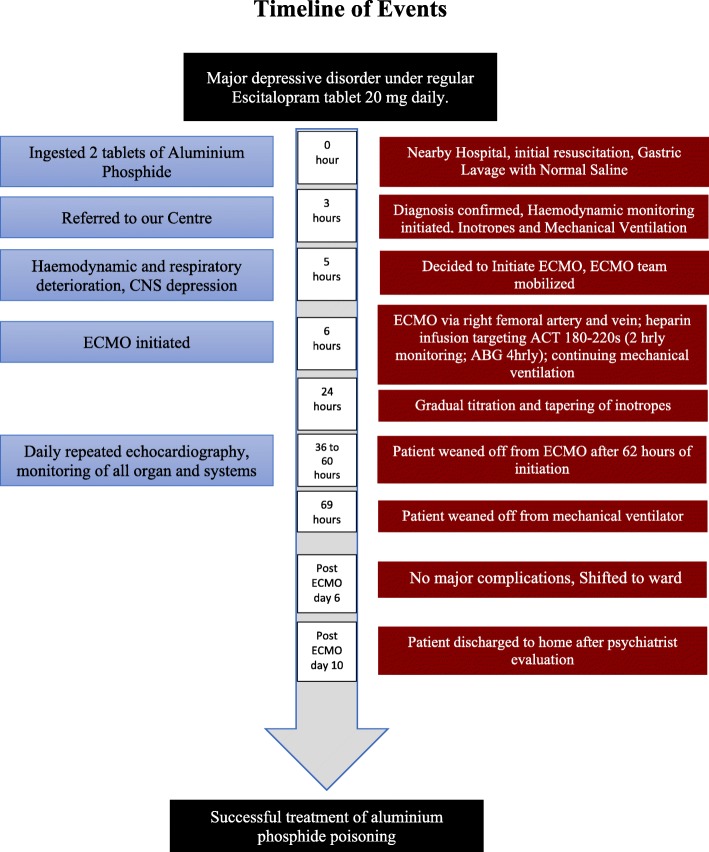
Timeline. *CNS* central nervous system, *ECMO* extracorporeal membrane oxygenation

An echocardiography done the next day showed significant improvement in cardiac status with LVEF reaching 35%. Her general condition, consciousness level, hemodynamic stability, and ventilatory parameters, especially peak airway pressure, also showed significant improvement over the next 3 days and we were able to stop all inotropic supports by ECMO day 2. She was finally weaned off the ECMO on day 3 and extubated the next day. At all times during her stay in ICU, although she showed signs of myocardial dysfunction and respiratory failure along with depression of mental status, her renal and liver function tests remained within normal range. A psychiatric evaluation was done while she was in ICU. After observation in ICU for 2 more days, she was discharged to ward with stable vital signs. She stayed in wards for 4 more days and was then finally discharged to home to have follow-up later and scheduled psychiatric consultation and adjustment of medications accordingly (timeline; Fig. 1).

## Discussion

A1P is a common suicide agent especially in countries like Nepal, India, and Iran [2–4]. Used for various domestic and agricultural purposes, this agent is readily available in the market. Accidental inhalation of A1P in fields and often suicidal ingestion of this agent by persons who are depressed or suffering from psychiatric illnesses [8] result in rapid deterioration in organ function. The deadly PH_3_ gas that is generated as a result of its interaction with environmental moisture or acid in the stomach results in inhibition of the cytochrome oxidase enzyme system in mitochondria due to which the body is unable to utilize oxygen for aerobic metabolism [9]. The ultimate dependence on anaerobic metabolism results in significant lactate accumulation and severe metabolic acidosis. In the major organ systems, myocardial dysfunction and respiratory failure lead to hypotension, hypoxemia, and ultimately death. Due to the unavailability of specific antidotes, several agents have been tried but none have been effective in its management and the mortality rate remains as high as 80–90% [10], although it varies depending on the amount consumed or inhaled. The only consistently promising treatment option for this poisoning has been ECMO. In this therapy, a “time-buy” approach is utilized in which the cardiovascular and respiratory systems are supported until the poison is eliminated from the body mainly by the kidneys. This approach to AlP poisoning is so effective that in one study from India, the clinicians were able to reduce the mortality rate from 88% in untreated cases to 33% when ECMO was used [11].

The significant difference in the management of AlP poisoning with ECMO in our case was its relatively early initialization before trying other treatment options. Due to the sheer amount of risk involved with ECMO, the usual practice of resorting to ECMO as an ultimate therapy when other therapies fail is common. However, deviation from that norm was necessary in our case for some specific reasons. First, unlike other cases of rodenticide poisoning where the population group is relatively young with healthy organ systems, our patient was a sexagenarian and did not have a good physiologic reserve and it was necessary to support her early given that her baseline echocardiography was not good. Second, as our center is well equipped with cardiovascular and good critical care unit backup, we were comfortable enough to start the therapy within a short period of time which ultimately would have helped our patient with much needed support. However, during the course of treatment the most pertinent question that we often asked ourselves and sought to find the answer to was: How long should we run ECMO? There is no definite answer to this question, however. Since the use of ECMO is indicated mostly for reversible pathologies, the assurance of the treatment of the primary pathology is probably the most important time when ECMO can be stopped. In our case, for example, the time taken for the poison to be eliminated from our patient’s body was the most appropriate time for weaning her off of ECMO. However, this cannot be objectively determined. Therefore, we resorted to methods of indirect tests such as daily echocardiography screening, chest X-rays, and daily physical examination including assessment of our patient’s consciousness level to determine the appropriate time for weaning off of ECMO. To the best of our knowledge, there are no set criteria or guidelines to guide us in this process and this weaning process remains at the discretion of the treating physicians.

The use of ECMO, however, is not only limited to poisoning. Veno-venous ECMO is designed to provide respiratory support especially in conditions like acute respiratory distress syndrome (ARDS)-associated refractory hypoxemia. In this technique, blood is removed from the venous side and pumped back into it, however, this does not provide hemodynamic support. In veno-arterial ECMO, blood is pumped from the venous side to the arterial side. In the process of doing this, it provides both gas exchange as well hemodynamic support [12]. The setup, however, for both these types of ECMO is similar. Blood movement across the circuit and into the patient’s large vessels is made possible by an external pump that forces the blood through a membrane oxygenator allowing gas exchange and carbon dioxide (CO_2_) removal in the process. Although the benefits of ECMO far outweigh the complications like hemorrhage and infection, with which it is most commonly associated, a consultation with an ECMO specialist or perfusionist is mandatory as the operation of these devices varies considerably across different models.

## Conclusions

Although the use of ECMO in AlP poisoning in other parts of the world is not new, this is a new experiment for us. Despite its availability, ECMO was never used for this purpose before in Nepal which we believe was due to hesitation because of fear about its success. In a country where considerable numbers of people die each year simply because of the result of their momentary decision to end their lives and who, otherwise, would have lived a productive life, there seems to be a possibility to reverse those reversible changes; this success surely opens doors to more opportunities. This simple yet novel approach to AlP treatment success in our center will certainly provide a much needed impetus to the flourishing of such uses in other centers as well and we hope to see many more lives saved in this mountainous country.

## Abbreviations

ABG: Arterial blood gas
ACT: Activated clotting time
AlP: Aluminum phosphide
ARDS: Acute respiratory distress syndrome
BP: Blood pressure
CO_2_: Carbon dioxide
ECG: Electrocardiogram
ECMO: Extracorporeal membrane oxygenation
ER: Emergency room
FiO_2_: Fraction of inspired oxygen concentration
GCS: Glasgow Coma Scale
HCO_3_: Bicarbonate
ICU: Intensive care unit
LVEF: Left ventricular ejection fraction
PaCO_2_: Partial pressure of carbon dioxide in blood
PaO_2_: Partial pressure of oxygen in blood
PEEP: Positive end-expiratory pressure
PH_3_: Phosphine
RR: Respiratory rate
